# Eruptive halo nevi after melanoma excision

**DOI:** 10.1016/j.jdcr.2026.05.066

**Published:** 2026-06-06

**Authors:** Vera Wang, Kevin Mai, Catherine Tawfik, Sabiha Uddin, Alexandra Flamm, Ronald Brancaccio

**Affiliations:** a Western University of Health Sciences College of Osteopathic Medicine of the Pacific, Pomona, California; b St Barnabas Hospital - Department of Dermatology, Bronx, New York; c Ronald O Perelman Department of Dermatology, New York University Grossman School of Medicine, New York

**Keywords:** eruptive halo nevi, excision, halo nevi, melanoma

Halo nevi are benign melanocytic nevi characterized by a central pigmented lesion with a surrounding well-defined area of depigmentation, creating a “halo” effect. These most commonly affect children and young adults and typically appear on the trunk, possibly from trauma or sunburn.^1^ Halo nevi likely occur via an immune-mediated process in which CD8^+^ T-lymphocytes target and destroy melanocytes in both the nevus and the surrounding skin. Thus, halo nevi first begin with the initial appearance of the halo, followed by progressive involution and possible complete regression of the central nevus, and eventual persistence or enlargement of the depigmented area.^2^ Histopathologically, the halo region often shows lymphocytic infiltration and loss of melanocytes, and the central nevus demonstrates features of regression.^1^

Halo nevi can occur with congenital nevi and, rarely, melanoma. A case series by Lorentzen et al^3^ reported that adults with eruptive or multiple halo nevi may have a higher prevalence of melanoma or other malignancies compared to the general population, suggesting that the sudden appearance of multiple halo nevi in adults warrants thorough evaluation for underlying malignancy.^4^ Overall, this is a rarely reported phenomenon but with potential for serious associations. We present a case of a 36-year-old man with eruptive halo nevi following a melanoma excision.

## Case report

**Fig 1:**
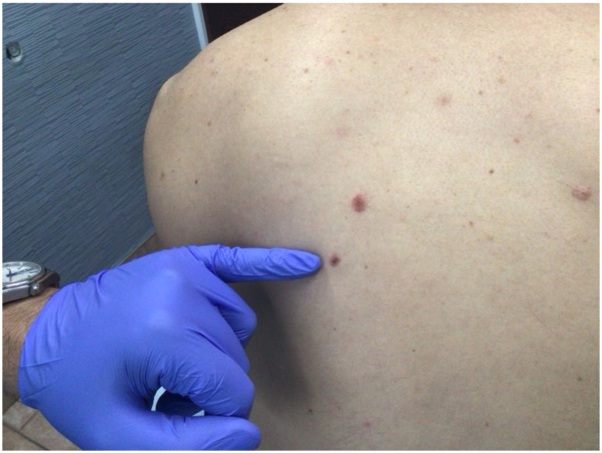
Melanoma biopsied on the upper middle portion of the left side of the back. The nevus superiomedial to the melanoma did not initially exhibit halo nevus features.

**Fig 2:**
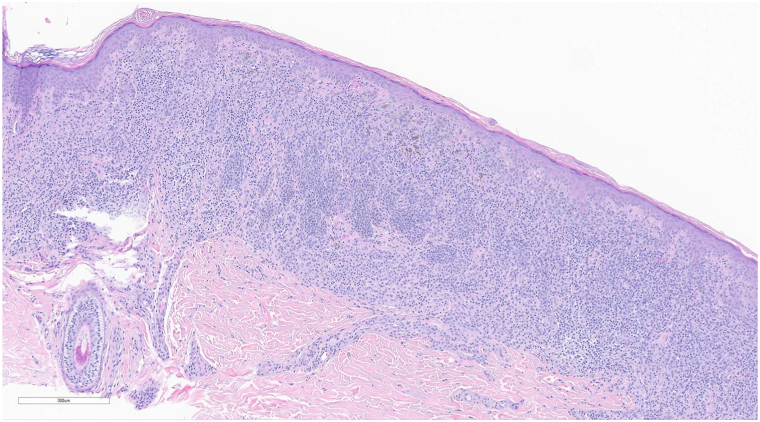
Histopathology of melanoma excision demonstrating asymmetric proliferation of melanocytes with enlarged hyperchromatic nuclei and scattered mitotic figures. (Hematoxylin-eosin stain; original magnification: ×10.)

**Fig 3:**
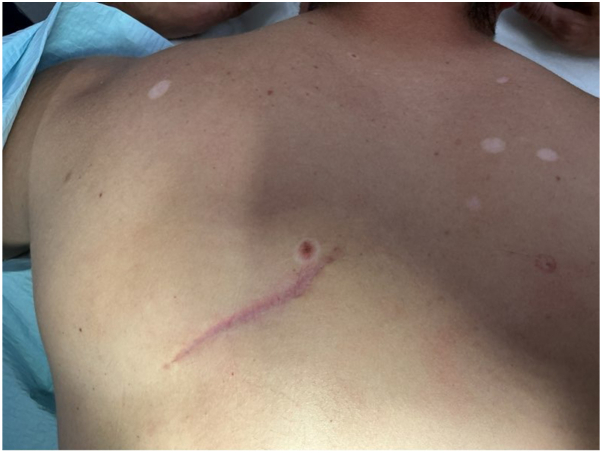
Development of halo nevi from pre-existing nevi in the periphery of the melanoma excision site.

Our patient is a 36 year-old-man with a paternal family history of melanoma and no personal history of any cancer who presented for a full body skin examination. A concerning pigmented lesion was biopsied on the upper middle portion of the left side back with the biopsy report returning as a melanoma of 0.8 mm thickness without any ulceration (Fig 1). On pathology, an asymmetric proliferation of melanocytes with many of the melanocytes having enlarged hyperchromatic nuclei and scattered mitotic figures were noted (Fig 2). There was also an associated brisk lymphocytic inflammatory infiltrate seen with the specimen. The lesion was subsequently excised with negative margins. Left axillary sentinel lymph node biopsies were negative for any melanoma as well. In a follow-up 3 months afterward, it was noted that the other nevi on the back had developed into halo nevi (Fig 3). The patient denied any history of any halo nevi or skin cancer, and no sign of recurrence was observed at the melanoma excision site.

## Discussion

Halo nevi can develop after certain medical therapies, particularly after the use of immune checkpoint inhibitors or targeted therapies. These induce immune activation against melanocytic antigens, enhancing cytotoxic T-cell responses and destruction of melanocytes. The appearance of halo nevi during immunotherapy or targeted therapy is considered an immune-related event and is associated with a favorable prognosis and robust antitumor response in melanoma patients.^5^ Melanoma-associated hypopigmentation with these treatments supports the immunologic theory of halo nevi pathogenesis.

Halo nevi are benign; however, association with malignancies should be considered. Lorentzen et al^3^ published a case series of 16 patients with eruptive halo nevi, 50% of whom had malignancies including melanoma, papillary thyroid carcinoma, and carcinoid tumor (Table I).^6^ The incidence of melanoma in this population was 995 times higher than expected for their age and sex (95% CI: 262-1.562), and incidences of papillary thyroid cancer and cancers overall were 247 and 113 times higher, respectively.^3^ This supports an association between melanoma and eruptive halo nevi, prompting cautious evaluation of patients with this condition.

**Table I:** Previously reported cases of melanoma associated with halo nevi

Publication	Age, y	Sex	Halo nevi found before or after melanoma excision	Time to halo nevi eruption, if after excision
Epstein et al^6^	30	M	Before	–
	32	M	After	1 wk
	25	M	After	2 wk
	55	M	After	2 wk
	56	F	After	Unclear
Lorentzen et al^3^	37	F	Before	–
	54	F	Before	–
	49	F	Before	–
	60	M	Before	–
	37	F	Before	–
	53	F	Before	–
	33	F	Before	–
	29	F	Before	–

F, female; M, male.

Eruptive halo nevi also have various nonmalignant associations. De Giorgi et al^7^ report a possible case in a 35-year-old-man related to SARS-CoV-2 vaccination, and Pastukhova and Ghazawi^8^ report a case in a 37-year-old woman 4 weeks after testing positive for SARS-Cov-2 infection. Autoimmune diseases such as vitiligo and Hashimoto thyroiditis, which are linked to immune-mediated melanocyte destruction, have been observed in patients with halo nevi.^1^ UV light exposure has also been proposed as an instigating factor in halo nevi development.^9^ Various neoplastic, immune, and systemic processes should be considered when evaluating patients with eruptive nevi.

The rates of eruptive halo nevi occurring after melanoma excision are not well-quantified given its rarity. The only published cases are from Epstein et al,^6^ who reported four patients with melanoma who received resection of primary cutaneous melanoma and afterwards developed halo nevi (Table I). Three patients had several to many halo nevi and two had a single halo nevus, with a mean age of 39.6 years. Timelines were specified for three patients, with their halo nevi appearing 1 to 2 weeks after excision.^6^ Our patient, in contrast, was found to have several halo nevi 3 months after excision, emphasizing the importance of follow-up. Theories proposed include a traumatic response or possible immune response triggered by melanocyte-related antigens, which would correspond with the brisk inflammatory infiltrate reported in the biopsy findings. Given cases of eruptive halo nevi presenting in patients years following a melanoma diagnosis, there is potential utility of further evaluation for secondary malignancies. Although the incidence of secondary malignancies was found to be higher than expected, further investigation and randomized trials are needed to adequately define further investigative approaches. Given the scarcity of other literature describing melanoma excision-related halo nevi and its unclear pathogenesis, more patients should be monitored for this phenomenon.

## Conflicts of interest

None disclosed.
